# Supplementary material for: Restoring confidence in return to work: A qualitative study of the experiences of persons with exhaustion disorder after a dialogue-based workplace intervention
Source: PLoS One. 2020 Jul 31;15(7):e0234897. doi: 10.1371/journal.pone.0234897 (PMC7394387; doi:10.1371/journal.pone.0234897)
Supplement: S1 Appendix — (PDF) [file pone.0234897.s001.pdf]

Interview with the employee before the convergence dialogue meeting

1. What expectations/concerns do you have – for this meeting?<br>– for work rehabilitation?	
2. Can you describe your work? (tasks, working hours)	
3. Do you use good practices at work? (for example cognitive or procedures)<br>– what practices?	☐ Completely<br>☐ Partly<br>☐ Not at all
4. To what extent do you think that your sick leave is caused by conditions at work?<br>– what conditions?	☐ Completely<br>☐ Partly<br>☐ Not at all
5. To what extent do you think that your sick leave is caused by circumstances outside your work?<br>– what conditions?	☐ Completely<br>☐ Partly<br>☐ Not at all
6. Has your employer/supervisor done something to facilitate your work situation before or during the current sick leave?<br>– if yes, what?	☐ Yes<br>☐ Partly<br>☐ No
7. Had you tried to bring about any changes before the actual sick leave?<br>– if yes, which ones?	☐ Yes<br>☐ Partly<br>☐ No
8. Can you describe what support you receive from your employer/supervisor as well as your colleagues?	
9. What tasks (work/leisure) can you currently perform without major difficulties?	
10. What tasks (work/leisure) do you currently avoid or find difficult to perform due to your problems?	
11. What are your own suggestions for returning to work?	
12. How important is it for you to get back to work/increase your working hours, on a scale of 0-10? (where 10 is very important)	Points:
13. What confidence do you have of success in getting back to work/increase your working hours on a scale of 0-10? (where 10 is the highest confidence of success)	Points:
14. How ready are you to get back to work/increase your working hours on a scale of 0-10? (where 10 is completely ready)	Points:
15. Homework before the next visit/contact/meeting:	
16. What has been said in confidence:	

Interview with the employer/supervisor before the convergence dialogue meeting

1. What expectations/ concerns do you have – for this meeting?<br>– for work rehabilitation?	
2. To what extent do you think that the employee's sick leave is caused by conditions at work?<br>– What conditions?	☐ Completely<br>☐ Partly<br>☐ Not at all<br>☐ Don't know
3. To what extent do you think that the employee's sick leave is caused by circumstances outside of work?<br>– What conditions?	☐ Completely<br>☐ Partly<br>☐ Not at all<br>☐ Don't know
4. As an employer/supervisor, did you do something to facilitate the work situation before the actual sick leave?<br>– If yes, what?	☐ Yes<br>☐ Partly<br>☐ No
5. Did the employee try to bring about any changes before the actual sick leave?<br>– If yes, which ones?	☐ Yes<br>☐ Partly<br>☐ No
6. Have you as an employer/supervisor done something to facilitate return to work?<br>– If yes, in what?	☐ Yes<br>☐ Partly<br>☐ No

Questions to the full-time or part-time employee

7. What tasks can the employee currently perform without major difficulties?	
8. What tasks does the employee avoid or find difficult to perform due to current problems?	
9. What are your own suggestions for returning to work?	
10. How important is it to you that the employee returns to work/ increases working hours on a scale of 0–10? (where 10 is very important)	Points:
11. What confidence do you have that the employee will succeed in returning to work/increasing work hours on a scale of 0–10? (where 10 is the highest confidence of success)	Points:
12. Do you work with a systematic work environment in your workplace?<br>– If yes, in which way?	☐ Yes<br>☐ No
13. Does your workplace have access to occupational healthcare?<br>– If yes, is a contact/action inserted?	☐ Yes<br>☐ No
14. What has been said in confidence:	

Summary of employee and employer perspectives before the convergence dialogue meeting

	Employee	Employer /supervisor
1. To what extent is sick leave caused by conditions at work?<br>– What conditions?	☐ Completely<br>☐ Partly<br>☐ Not at all	☐ Completely<br>☐ Partly<br>☐ Not at all<br>☐ Don't know
2. To what extent is sick leave caused by conditions outside work?<br>– What conditions?	☐ Completely<br>☐ Partly<br>☐ Not at all	☐ Completely<br>☐ Partly<br>☐ Not at all<br>☐ Don't know
3. Has the employer/supervisor done something to facilitate the work situation before the current sick leave?<br>– If yes, what?	☐ Yes<br>☐ Partly<br>☐ No	☐ Yes<br>☐ Partly<br>☐ No
4. Has the employee tried to bring about any changes before the current sick leave?<br>– If yes, which ones?	☐ Yes<br>☐ Partly<br>☐ No	☐ Yes<br>☐ Partly<br>☐ No
5. Has the employer done something to facilitate return to work?<br>– If yes, in what?	☐ Yes<br>☐ Partly<br>☐ No	☐ Yes<br>☐ Partly<br>☐ No
6. What tasks can the employee currently perform without major difficulties?		
7. What tasks does the employee avoid or find difficult to perform due to current problems?		
8. The employee’s and employer’s proposals for return to work		
