# Supplementary material for: Restoring confidence in return to work: A qualitative study of the experiences of persons with exhaustion disorder after a dialogue-based workplace intervention
Source: PLoS One. 2020 Jul 31;15(7):e0234897. doi: 10.1371/journal.pone.0234897 (PMC7394387; doi:10.1371/journal.pone.0234897)
Supplement: S2 Appendix — (PDF) [file pone.0234897.s002.pdf]

Intervjuguide – strukturerad trestegs intervjumodell

Personintervju inför dialogsamtal

1. Vilka förväntningar/farhågor har du - inför detta samtal? – inför arbetsrehabilitering?	
2. Kan du beskriva ditt arbete? (arbetsuppgifter, arbetstider)	
3. Använder du dig av goda rutiner på arbetet? (se ex. kognitiv- och belastningsergonomi)<br>- vilka rutiner?	☐ Helt<br>☐ Delvis<br>☐ Inte alls
4. I vilken utsträckning anser du att sjukskrivningen är orsakad av förhållanden *på ditt arbete?*<br>- vilka förhållanden?	☐ Helt<br>☐ Delvis<br>☐ Inte alls
5. I vilken utsträckning anser du att sjukskrivningen är orsakad av förhållanden *utanför ditt arbete?*<br>- vilka förhållanden?	☐ Helt<br>☐ Delvis<br>☐ Inte alls
6. Har din arbetsgivare/arbetsledare gjort något för att underlätta din arbetssituation *före* respektive *under* aktuell sjukskrivning?<br>- om ja, på vilket sätt?	☐ Ja<br>☐ Delvis<br>☐ Nej
7. Har du *före* aktuell sjukskrivning själv försökt få några förändringar till stånd?<br>- om ja, vilka?	☐ Ja<br>☐ Delvis<br>☐ Nej
8. Kan du beskriva vilket stöd du upplever från din arbetsgivare/arbetsledare samt dina kollegor?	
9. Vilka uppgifter (arbete/fritid) kan du idag utföra utan större hinder?	
10. Vilka uppgifter (arbete/fritid) har du idag svårt att utföra/undviker på grund av dina besvär?	
11. Vilka är dina egna förslag för återgång i arbete?	
12. Hur *viktigt* är det för dig att komma tillbaka till arbetet/öka i arbetstid på en skala 0-10?<br>(där 10 är jätteviktigt)	Poäng:
13. Vilken *tilltro* har du till att lyckas komma tillbaka till arbetet/öka i arbetstid på en skala 0-10?<br>(där 10 är högsta tilltro till att lyckas)	Poäng:
14. Hur *redo* är du att komma tillbaka till arbetet/öka i arbetstid på en skala 0-10?<br>(där 10 är fullt redo)	Poäng:
15. Hemuppgift att göra fram till nästa besök/kontakt/dialogsamtal:	
16. Vad har sagts i förtroende:	

Arbetsgivarintervju inför dialogsamtal

1. Vilka förväntningar/farhågor har du - inför detta samtal? – inför arbetsrehabilitering?	
2. I vilken utsträckning anser du att den anställdes sjukskrivning är orsakad av förhållanden *på arbetet?*<br>- vilka förhållanden?	☐ Helt<br>☐ Delvis<br>☐ Inte alls<br>☐ Vet ej
3. I vilken utsträckning anser du att den anställdes sjukskrivning är orsakad av förhållanden *utanför arbetet?*<br>- vilka förhållanden?	☐ Helt<br>☐ Delvis<br>☐ Inte alls<br>☐ Vet ej
4. Har du som arbetsgivare/arbetsledare gjort något för att underlätta arbetssituation *före* aktuell sjukskrivning?<br>- om ja, vad?	☐ Ja<br>☐ Nej
5. Har den anställde *före* aktuell sjukskrivning själv försökt få några förändringar till stånd?<br>- om ja, vilka?	☐ Ja<br>☐ Nej
6. Har du som arbetsgivare/arbetsledare gjort något för att underlätta *återgång* i arbete?<br>- om ja, på vilket sätt?	☐ Ja<br>☐ Delvis<br>☐ Nej
**Frågor att ställa om den anställde arbetstränar/arbetar hel- eller deltid:**<br><br>7. Vilka arbetsuppgifter kan den anställde utföra idag, utan större hinder?<br><br>8. Vilka arbetsuppgifter har den anställde svårt att utföra/undviker idag, på grund av sina besvär?	
9. Vilka är dina egna förslag för återgång i arbete?	
10. Hur *viktigt* är det för dig att den anställde kommer tillbaka till arbetet/ökar sin arbetstid på en skala 0-10? (där 10 är jätteviktigt)	Poäng:
11. Vilken *tilltro* har du till att den anställde lyckas komma tillbaka till arbetet/öka sin arbetstid på en skala 0-10? (där 10 är högsta tilltro till att lyckas)	Poäng:
12. Arbetar ni med systematiskt arbetsmiljöarbete (SAM) på er arbetsplats?<br>- om ja, på vilket sätt?	☐ Ja<br>☐ Nej
13. Har er arbetsplats tillgång till företagshälsovård?<br>- om ja, är kontakt/åtgärder insatta?	☐ Ja<br>☐ Nej
14. Vad har sagts i förtroende:	

Sammanfattning av person- och arbetsgivarperspektiv inför dialogsamtal

	**Person**	**Arbetsgivare**
1. I vilken utsträckning anses sjukskrivningen orsakad av förhållanden *på arbetet?*<br><br>- vilka förhållanden?	☐ Helt<br>☐ Delvis<br>☐ Inte alls<br>☐ Vet ej	☐ Helt<br>☐ Delvis<br>☐ Inte alls<br>☐ Vet ej
2. I vilken utsträckning anses sjukskrivningen orsakad av förhållanden *utanför arbetet?*<br><br>- vilka förhållanden?	☐ Helt<br>☐ Delvis<br>☐ Inte alls<br>☐ Vet ej	☐ Helt<br>☐ Delvis<br>☐ Inte alls<br>☐ Vet ej
3. Har arbetsgivaren gjort något för att underlätta arbetssituation *före* aktuell sjukskrivning?<br><br>- om ja, vad?	☐ Ja<br>☐ Nej	☐ Ja<br>☐ Nej
4. Har personen *före* aktuell sjukskrivning själv försökt få några förändringar till stånd?<br><br>- om ja, vilka?	☐ Ja<br>☐ Nej	☐ Ja<br>☐ Nej
5. Har arbetsgivaren gjort något för att underlätta *återgång* i arbete?<br><br>- om ja, på vilket sätt?	☐ Ja<br>☐ Delvis<br>☐ Nej	☐ Ja<br>☐ Delvis<br>☐ Nej
6. Vilka arbetsuppgifter kan den anställde utföra idag, utan större hinder?		
7. Vilka arbetsuppgifter har den anställde svårt att utföra/undviker idag, på grund av sina besvär?		
8. Parternas egna förslag för återgång i arbete?		
